# Procedural and Device Neutrality of Post-TAVI Renal Outcomes: A Multivariable Analysis of Valve Type, Size, and Anatomy

**DOI:** 10.3390/jcm15062175

**Published:** 2026-03-12

**Authors:** Rosa Alba Pugliesi, Shu Fon Muna, Andreas H. Mahken, Nour Maalouf, Georgios Chatzis, Jonas Apitzsch

**Affiliations:** 1 Department of Biomedicine, Neuroscience and Advanced Diagnostics (BiND), University of Palermo, Via del Vespro 129, 90127 Palermo, Italy; 2 Department of Urology, University Hospital OWL, Klinikum Bethel gGmbH, 33611 Bielefeld, Germany; shu-fon.muna@evkb.de; 3 Department of Diagnostic and Interventional Radiology, University Hospital of Marburg, 35043 Marburg, Germany; 4 Department of Diagnostic and Interventional Radiology, University Hospital of Tübingen, 72076 Tübingen, Germany; nourmaalouff@gmail.com; 5 Department of Cardiology, Angiology, and Intensive Care Medicine, University Hospital, Philipps University of Marburg, 35043 Marburg, Germany; chatzis@staff.uni-marburg.de; 6 Department of Radiology and Nuclear Medicine, Helios Hospital Pforzheim, 75175 Pforzheim, Germany; jonas.apitzsch@helios-gesundheit.de

**Keywords:** transcatheter aortic valve implantation, acute kidney injury, aortic valve diameter, creatinine, eGFR change

## Abstract

**Background:** Renal dysfunction remains a frequent complication after transcatheter aortic valve implantation (TAVI). Although contrast exposure and baseline renal impairment are established risk factors, the influence of structural valve characteristics, including valve diameter and prosthesis platform, on early renal outcomes is not well defined. This study evaluated whether valve size and valve platform independently affect early post-procedural renal function. **Methods:** This retrospective cohort study included 410 consecutive patients undergoing TAVI between 2018 and 2021 with complete pre- and post-procedural renal biomarker data. Of these, 371 patients with complete covariate data were analyzed in multivariable models. Serum creatinine and estimated glomerular filtration rate (eGFR) were assessed within 72 h before and after TAVI. Renal function change was calculated as absolute differences. Acute kidney injury (AKI) was defined according to KDIGO criteria. Correlation analyses and multivariable linear and logistic regression models were performed. **Results:** Median valve diameter was 26 mm (IQR 26–29). Renal function remained largely stable, with a median creatinine change of −0.06 mg/dL and median eGFR change of +4.0 mL/min/1.73 m^2^. Valve diameter was not associated with creatinine change (ρ = −0.047, *p* = 0.330) or eGFR change (ρ = 0.053, *p* = 0.278). KDIGO-defined AKI occurred in 56 patients (13.7%) and did not differ by valve platform (*p* = 0.719) or diameter tertiles (*p* = 0.204). **Conclusions:** Valve diameter and platform were not independently associated with early renal outcomes after TAVI. Baseline renal function and contrast exposure were the principal determinants of post-procedural renal trajectory.

## 1. Introduction

In patients with intermediate or high surgical risk, transcatheter aortic valve implantation (TAVI) has become the standard-of-care treatment for severe aortic stenosis, serving as an alternative to traditional surgical aortic valve replacement (SAVR) [1,2]. As valve design, imaging, and implantation techniques have improved, even lower-risk patients can achieve outcomes comparable to SAVR, enhancing survival and quality of life [3].

These technological and procedural advances have made TAVI more accessible to more patients, resulting in widespread use [4]. Despite these advances, acute kidney damage (AKI) remains a prominent consequence of TAVI, especially in elderly or CKD patients, with iodinated contrast exposure further exacerbating renal risk [5]. Kidney injury following TAVI results from a complex interplay of patient-specific factors (including baseline renal function, comorbidities, and vascular status), procedural factors (such as contrast volume, access route, and procedural duration), and device-related characteristics (including prosthesis type, size, and deployment mechanics) [6]. Even in the absence of contrast exposure, recent studies indicate that transient renal dysfunction may occur, presumably as a result of altered blood flow dynamics and microembolization of calcific debris during valve deployment [7]. These results suggest that renal outcomes may be impacted by the mechanical properties of prostheses, despite the implementation of strategies to reduce contrast use and maintain hemodynamic stability [8].

Nevertheless, the impact of valve design, prosthesis size, and leaflet biomechanics on post-TAVI renal outcomes remains insufficiently explored in the literature. 

Valve characteristics, including prosthesis type, valve size, and leaflet configuration, can influence post-implantation hemodynamics, thrombogenicity, and flow patterns, potentially leading to microembolization and renal perfusion disruptions [9]. The size and geometry of the aortic annulus directly influence the selection of a prosthesis, the method of deployment, and the difficulty of the intervention in TAVI planning [10]. Paravalvular leak or annular perforation may result from a mismatch between the prosthesis and annular anatomy, which could potentially compromise renal perfusion and hemodynamic stability [11].

Larger prosthetic valves have been linked to an elevated risk of acute kidney injury (AKI) in numerous studies, potentially as a result of the prolonged fluoroscopy times and higher contrast volumes necessary during implantation [12,13]. In contrast, some studies suggest that baseline renal function and overall comorbidity burden are more reliable predictors of post-TAVI renal outcomes than prosthesis size, with patients who have pre-existing renal dysfunction, diabetes, or other chronic conditions remaining at higher risk regardless of valve characteristics [14,15]. Furthermore, evidence from large multicenter registries indicates that balloon-expandable and self-expanding valves exhibit comparable renal safety profiles, supporting the notion that prosthesis type may have a neutral effect on post-procedural renal risk [2].

This evidence raises questions about whether concerns regarding prosthesis size and type are overemphasized, or whether patient-related factors and procedural techniques play a more decisive role in determining renal outcomes [16]. Transvalvular gradients and prosthetic valve expansion are also influenced by annular severity and asymmetry, while leaflet calcification may further compromise renal perfusion and contribute to postoperative kidney dysfunction [17,18]. Although computed tomography (CT) provides a comprehensive assessment of aortic anatomy and simplifies the selection of a prosthesis, the correlation between CT and renal changes following TAVI remains incomplete [19]. Clarifying the determinants of renal function after TAVI may enable improved procedural planning, refined risk stratification, and more effective prevention of kidney injury [20].

This study seeks to clarify the relationship between prosthetic valve characteristics and early renal outcomes following TAVI by specifically evaluating the impact of valve diameter and type on short-term changes in renal function. We aim to determine whether these device-related parameters independently influence post-procedural renal function after adjustment for baseline kidney status and procedural hemodynamic variables. By isolating the contribution of prosthesis-related factors from patient comorbidities and procedural complexity, this study directly examines the role of TAVI-specific features in the development of acute kidney injury.

Understanding these interactions is essential for identifying potentially modifiable procedural determinants of renal impairment. As TAVI continues to expand into lower-risk populations, even modest differences in extracardiac complications such as renal dysfunction may meaningfully affect recovery trajectories, length of hospitalization, and long-term outcomes. Therefore, clarifying whether valve selection and sizing contribute to renal risk has direct implications for device choice, procedural planning, and comprehensive peri-procedural risk assessment in contemporary TAVI practice.

## 2. Material and Methods

### 2.1. Study Design and Population

The study protocol was approved by the institutional review board, and written informed consent was obtained from all participants in accordance with the Declaration of Helsinki and applicable institutional regulations.

We conducted a retrospective cohort study including all consecutive patients who underwent TAVI from January 2018 to December 2021 at a tertiary cardiovascular center. Patients were identified through the institutional TAVI registry, which prospectively collects demographic, clinical, and procedural data. Extracted variables included age, gender, valve type, valve diameter, and procedural characteristics.

### 2.2. Inclusion and Exclusion Criteria

Of the total TAVI procedures performed during the study period, 410 patients had complete pre- and post-procedural renal biomarker data and were included in the primary renal outcome analysis. Among these, 371 patients had complete data for all covariates required for multivariable regression modeling and therefore constituted the final analytic cohort for adjusted analyses. Inclusion criteria comprised successful completion of the TAVI procedure, availability of pre- and postprocedural renal biomarker measurements (serum creatinine and estimated glomerular filtration rate [eGFR]), and documented left ventricular ejection fraction (EF) as a principal covariate.

**Figure 1:**
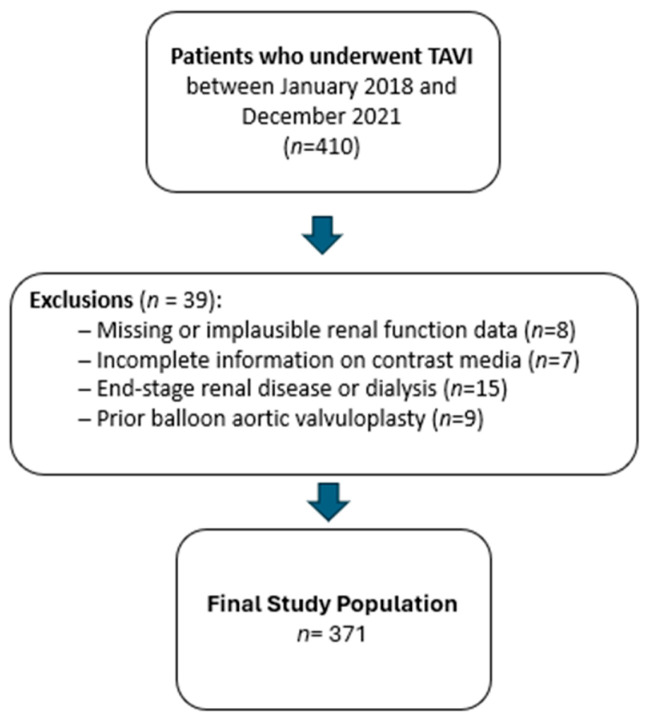
Study Flowchart. Overview of participant recruitment, interventions, data collection, and analysis process in the study.

Patients were excluded if they had missing pre- or post-TAVI creatinine or eGFR values, lacked data on contrast medium volume used during computed tomography or the TAVI procedure, exhibited implausible contrast dosing (e.g., 0.1 mL or 10 mL), had undergone prior balloon aortic valvuloplasty, or had end-stage renal disease requiring dialysis. The final cohort included 210 women (56.7%) and 161 men (43.3%) (Figure 1).

### 2.3. Renal Function Assessment

Renal function was evaluated through serial measurements of serum creatinine (mg/dL) and estimated glomerular filtration rate (eGFR, mL/min/1.73 m^2^), obtained within 72 h before and within 72 h after the TAVI procedure. The estimated Glomerular Filtration Rate (eGFR) was determined utilizing the Modification of Diet in Renal Disease (MDRD) equation [21]. Changes in renal function were measured as the absolute difference between post-procedural and pre-procedural values:

ΔCreatinine = Post-TAVI creatinine − Pre-TAVI creatinine

ΔeGFR = Post-TAVI eGFR − Pre-TAVI eGFR

Acute kidney injury (AKI) was defined according to Kidney Disease: Improving Global Outcomes (KDIGO) criteria as an increase in serum creatinine of ≥0.3 mg/dL within 48 h or ≥1.5 times baseline within 7 days [22,23,24].

Changes in eGFR were analyzed as secondary continuous outcomes but were not used to define KDIGO-based AKI [25,26].

In response to current guideline recommendations favoring the CKD Epidemiology Collaboration (CKD-EPI) equation, a prespecified sensitivity analysis was performed using CKD-EPI-derived eGFR values. Results were consistent with the primary MDRD-based analysis, and no significant association between valve characteristics and renal outcomes was observed.

To identify independent predictors of worsening renal function, multivariable regression models were developed, adjusting for clinically relevant covariates such as age, gender, baseline eGFR, contrast volume, access route (transfemoral versus alternative access), valve type (balloon-expandable versus self-expanding), valve size, procedural duration, utilization of rapid ventricular pacing, and peri-procedural hemodynamic instability, in accordance with previous studies assessing renal outcomes following TAVI [27,28,29].

### 2.4. Statistical Analysis

Descriptive statistics for the available variables were computed, and continuous data are presented as mean ± standard deviation or median and IQR based on the nature of distribution as determined by the Shapiro–Wilk test. Both creatinine change and eGFR change variables were not of normal distribution (*p* < 0.001); therefore nonparametric Spearman rank correlation was applied to assess the relations between valve diameter and renal function changes. Differences by gender were compared using the Wilcoxon rank-sum test.

To control for confounding potential, two multivariable linear regression models were developed, and creatinine change and eGFR change used as outcomes. The predictor variables entered were valve diameter, age, gender, and baseline renal function (creatinine or eGFR). Robust regression with Huber’s M-estimation was performed to control for eGFR change outliers and non-normality.

Finally, a binary logistic regression model was constructed to identify independent predictors of AKI, with valve diameter, age, gender, and baseline eGFR as covariates. Due to substantial missing data, contrast volume and access route could not be included in the final multivariable logistic regression model. Statistical significance was defined as a two-tailed *p*-value less than 0.05. Statistical analyses were performed using R software (version 4.5.1).

## 3. Results

### 3.1. Descriptive Analysis

A total of 410 patients were included in the primary renal outcome analysis. Of these, 371 patients (56.7% female) with a mean age of 81.8 ± 5.9 years had complete covariate data and were included in multivariable modeling. The median valve diameter was 26 mm (IQR 26–29), with a broad size range reflecting real-world clinical practice. Renal function demonstrated minimal median change post-TAVI, with slight improvement in eGFR (+4.0 mL/min/1.73 m^2^) and small decrease in serum creatinine (−0.06 mg/dL), suggesting generally stable kidney function following intervention (Table 1).

These baseline descriptive statistics provide the measure against which to investigate associations between valve size and renal outcomes.

### 3.2. Correlation and Group Comparisons

Spearman rank correlation analysis identified no statistically significant relationship between valve diameter and renal function change. Specifically, the correlation coefficient between valve diameter and creatinine change was −0.047 (*p* = 0.330), and between valve diameter and eGFR change was +0.053 (*p* = 0.278), indicating weak and non-significant relationships (Table 2).

When comparing renal outcomes between genders, a non-significant trend toward greater creatinine increase in men was observed (*p* = 0.051).

**Figure 2:**
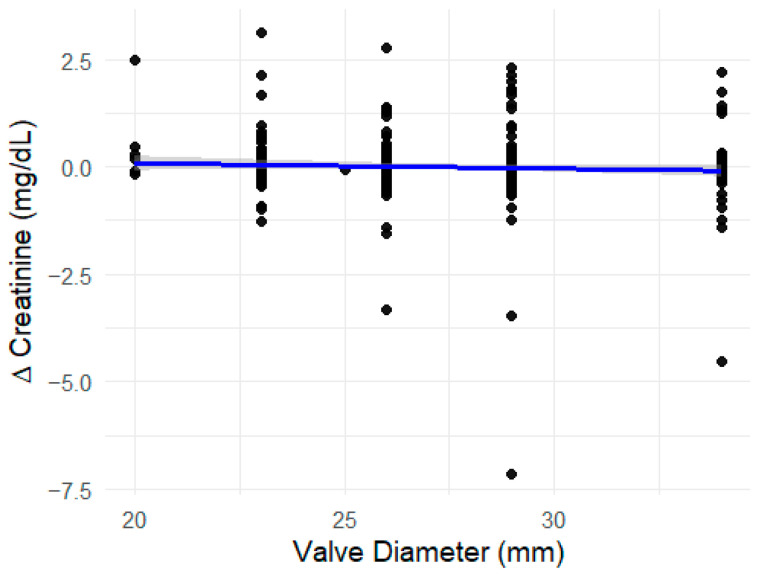
Valve Diameter and Change in Serum Creatinine After TAVI. Scatter plot showing the relationship between valve diameter (mm) and the change in serum creatinine (Δ Creatinine, mg/dL) following TAVI. Each point represents an individual case. A blue linear regression line with a 95% confidence interval (shaded area) is overlaid to visualize the trend. The nearly flat regression line indicates no significant linear relationship between valve size and postoperative change in renal function, in agreement with the non-significant Spearman correlation result.

Figure 2 illustrates the scatter plot of valve diameter against creatinine change, supplemented by a linear regression smoothing line. The plot visually confirms the absence of a meaningful linear association between valve size and postprocedural creatinine variation.

### 3.3. Multivariable Regression Analysis

To adjust for potential confounders, multivariable linear regression models were constructed with change in creatinine and change in eGFR as dependent variables (Table 3).

Valve diameter did not emerge as a significant predictor in either model: in the creatinine model including contrast exposure, valve diameter remained non-significant (β = −0.0105, *p* = 0.313). In contrast, baseline creatinine was strongly and inversely associated with postprocedural creatinine change (β = −0.275, *p* < 0.001).

Importantly, contrast volume demonstrated an independent positive association with creatinine increase (β = 0.00101 per mL, *p* = 0.043), indicating that higher contrast exposure was associated with greater postprocedural renal impairment.

### 3.4. KDIGO-Defined Acute Kidney Injury

AKI was defined according to Kidney Disease: Improving Global Outcomes (KDIGO) criteria as an increase in serum creatinine ≥0.3 mg/dL or ≥1.5 times baseline within 72 h after TAVI. Overall, AKI occurred in 56 of 410 patients (13.7%) in the analyzable cohort with complete renal data.

#### 3.4.1. AKI by Valve Platform

**Figure 3:**
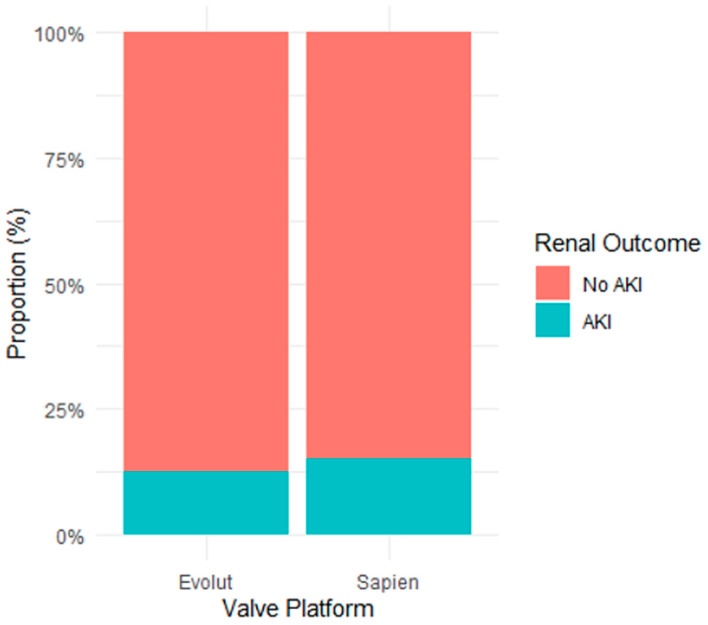
Incidence of KDIGO-Defined Worsening of Renal Function by Valve Platform. Stacked proportional bar chart illustrating the incidence of KDIGO-defined acute kidney injury (AKI) according to valve platform. The proportion of AKI did not differ significantly between balloon-expandable and self-expanding valves (*p* = 0.719).

Among balloon-expandable valves (Sapien), AKI occurred in 23 of 151 patients (15.2%). Among self-expanding valves (Evolut), AKI occurred in 33 of 259 patients (12.7%) (Figure 3). 

One case labeled as “n.a.” (n = 1) had no AKI event and was excluded from comparative platform analysis.

There was no statistically significant difference in AKI incidence between platforms (χ^2^ = 0.66, *p* = 0.719).

#### 3.4.2. AKI by Valve Diameter Tertiles

When stratified by valve diameter tertiles (n = 410), AKI occurred in 20 of 137 patients (14.6%) in tertile 1, 23 of 137 patients (16.8%) in tertile 2, and 13 of 136 patients (9.6%) in tertile 3.

No statistically significant difference in AKI incidence was observed across tertiles (χ^2^ test *p* = 0.204; Fisher’s exact *p* = 0.195) (Table 4).

Valve platforms were categorized as balloon-expandable (Sapien) or self-expanding (Evolut). One entry labeled “n.a.” (n = 1) was excluded from platform comparison. Analyses were performed on patients with complete data for the respective variables.

### 3.5. Multivariable Logistic Regression Predicting AKI

A multivariable logistic regression model was constructed to identify independent predictors of KDIGO-defined AKI. The model included valve diameter, age, gender, baseline renal function (eGFR before TAVI), valve platform, and contrast volume.

Valve diameter was not independently associated with AKI (OR = 0.96, 95% CI 0.83–1.09, *p* = 0.505). Age (OR = 0.99, *p* = 0.595) and gender (OR = 1.26, *p* = 0.550) were likewise not significantly associated with AKI. Valve platform showed no independent association (OR = 0.89, *p* = 0.784).

Baseline eGFR was a significant independent predictor (OR = 0.96 per mL/min/1.73 m^2^ increase, 95% CI 0.94–0.98, *p* < 0.001), indicating that lower preprocedural renal function was associated with higher AKI risk.

Contrast volume was also independently associated with AKI (OR = 1.006 per mL increase, 95% CI 1.002–1.011, *p* = 0.003). This corresponds to approximately a 6% increase in odds of AKI per additional 10 mL of contrast administered.

The model demonstrated improved explanatory performance compared with unadjusted analyses (AIC = 298.14).

### 3.6. Valve Platform Analysis

Valve platform analysis was restricted to the two primary prosthesis types used in this cohort: balloon-expandable (Sapien) and self-expanding (Evolut) systems. One patient labeled as “n.a.” was excluded from comparative analysis.

Across unadjusted comparisons and multivariable modeling, valve platform was not associated with early postprocedural renal outcomes. The incidence of KDIGO-defined AKI was comparable between Sapien and Evolut systems, and platform type did not emerge as an independent predictor in logistic regression analysis.

## 4. Discussion

In this retrospective single-center cohort, no independent association was observed between valve diameter or valve platform and early (≤72 h) renal outcomes.

Across correlation testing, multivariable linear modeling, logistic regression, and interaction analysis, valve diameter and valve platform were not associated with creatinine change, eGFR change, or KDIGO-defined AKI. Baseline renal function emerged as the strongest independent predictor of post-procedural kidney response.

Renal outcomes were assessed only within 72 h, precluding evaluation of subacute (7–30 day) and long-term (≥90 day) renal trajectories. Residual confounding cannot be excluded due to the retrospective design and incomplete availability of detailed intraprocedural hemodynamic parameters.

Therefore, potential subacute (7–30 day) or chronic (≥90 day) renal trajectories, which may differ mechanistically from very early changes, could not be evaluated in the present analysis. Future prospective studies incorporating serial follow-up assessments are warranted.

These findings align with prior registry- and cohort-based studies emphasizing that clinical comorbidities, rather than structural valve characteristics, drive renal risk after TAVI [6,8,25,30]. A meta-analysis further supports that demographic and baseline clinical factors are more reliable predictors of post-TAVI renal complications than valve anatomy or procedural device attributes [31]. Mechanistically, renal perfusion may be more sensitive to hemodynamic stability, cardiac output, and microembolization risk than to prosthesis size or expansion dynamics [32,33]. Any theoretical influence of valve diameter or type on renal outcomes is likely attenuated by the overall success of TAVI in restoring cardiac function.

Consistent with previous studies, valve type did not affect short-term renal function. Patients receiving Evolut or Sapien prostheses showed comparable serum creatinine and eGFR changes, even after adjusting for valve diameter and demographic variables [34,35]. Observed gender-related differences in renal response were minimal and disappeared after accounting for baseline renal function, indicating that pre-existing kidney status, rather than gender, determines postprocedural vulnerability [36].

Several limitations warrant consideration. The retrospective design limits causal inference and may leave residual confounding despite multivariable adjustment. Renal function was assessed only within 72 h after TAVI, preventing evaluation of medium- or long-term kidney outcomes. Additionally, incomplete data on contrast volume and intra-procedural hemodynamics limited assessment of their potential influence on renal outcomes. Patients with end-stage renal disease on dialysis were excluded, restricting generalizability to this high-risk subgroup. Important intraprocedural variables such as rapid ventricular pacing duration and transient hemodynamic instability were not systematically available and therefore could not be incorporated into the final regression models.

Despite these limitations, this study provides clinically relevant insights: early post-TAVI renal outcomes are chiefly determined by baseline kidney function, not by valve size or type. These findings underscore the importance of preprocedural renal optimization and minimizing nephrotoxic exposures, rather than focusing solely on anatomical or device-related features, to guide risk stratification and procedural planning [37,38]. Future prospective studies incorporating biomarkers such as Neutrophil Gelatinase-Associated Lipocalin (NGAL) or cystatin C may help detect subclinical injury and further refine individualized renal risk assessment [39].

## 5. Conclusions

In this retrospective cohort with short-term follow-up, baseline renal function appeared to be the dominant determinant of early renal trajectory, while structural valve characteristics, including prosthesis diameter and platform, were not independently associated with early KDIGO-defined renal deterioration. These findings suggest that, in contemporary TAVI practice, optimization of patient-related risk factors may be more critical for early renal protection than prosthesis selection itself.

## Figures and Tables

**Table 1:** Descriptive Statistics of the Study Population. The table provides a comprehensive overview of the study population’s baseline characteristics and renal outcomes following transcatheter aortic valve implantation (TAVI). Continuous variables are presented as mean ± standard deviation (SD), median with interquartile range (IQR), and full range. Renal function was evaluated based on the difference in serum creatinine and estimated glomerular filtration rate (eGFR) measured 72 h before and after TAVI. These statistics establish the baseline context for subsequent analyses on the association between valve size and postprocedural renal function.

Variable	Mean ± SD	Median (IQR)	Range (Min–Max)
Age (years)	81.8 ± 5.9	83 (79–86)	60–100
Valve Diameter (mm)	27.7 ± 3.8	26 (26–29)	23–34
Change in Creatinine (mg/dL)	−0.013 ± 0.714	−0.06 (−0.20–0.09)	−7.14–3.14
Change in eGFR (mL/min/1.73 m^2^)	3.83 ± 15.79	4.0 (−4.0–13.0)	−71–60

**Table 2:** Spearman Correlation Between Valve Diameter and Renal Function. Results of the Spearman rank correlation analysis assessing the relationship between aortic valve diameter and changes in renal function following TAVI. Both serum creatinine change and eGFR change were analyzed. The correlation coefficients indicate a weak and statistically non-significant association between valve diameter and renal outcomes.

Outcome	Spearman’s ρ	*p*-Value
Creatinine change	−0.047	0.330
eGFR change	+0.053	0.278

**Table 3:** Multivariable Linear Regression Models. This table displays two multivariable linear regression models evaluating predictors of renal function change after TAVI. Model A examines changes in eGFR, while Model B focuses on serum creatinine change. Independent variables included valve diameter, age, gender, baseline renal function, and contrast volume. The preprocedural eGFR and creatinine levels were significant predictors of their respective outcomes, whereas valve diameter was not significantly associated with renal recovery in either model.

A. eGFR Change Model			
Predictor	β (Estimate)	Std. Error	*p*-Value
Valve diameter	0.175	0.237	0.460
Age	0.032	0.095	0.742
Gender (male)	1.681	1.782	0.346
eGFR before TAVI	−0.087	0.037	0.018 *
Adjusted R^2^ = 0.009			
B. Creatinine Change Model			
Predictor	β (Estimate)	Std. Error	*p*-value
Valve diameter	−0.0105	0.0104	0.313
Age	0.00047	0.00555	0.933
Gender (female vs. male)	−0.0235	0.0781	0.764
Creatinine before TAVI	−0.2750	0.0278	<0.001 *
Contrast volume (mL)	0.00101	0.00050	0.043 *
Adjusted R^2^: 0.203			
Overall model *p*-value: <2.2 × 10^−16^			

An asterisk (*) indicates a statistically significant predictor (*p* < 0.05).

**Table 4:** Incidence of KDIGO-Defined AKI.

Group	N	AKI n (%)	*p*-Value
Balloon-expandable (Sapien)	151	23 (15.2%)	
Self-expanding (Evolut)	259	33 (12.7%)	0.719
Valve diameter tertile 1	137	20 (14.6%)	
Valve diameter tertile 2	137	23 (16.8%)	
Valve diameter tertile 3	136	13 (9.6%)	0.204

## Data Availability

The data presented in this study are openly available in Pugliesi, Rosa Alba (2026). TAVI Dataset. figshare. Dataset. https://doi.org/10.6084/m9.figshare.31146352.
